# Hyperelastic Properties of Bamboo Cellulosic Fibre–Reinforced Silicone Rubber Biocomposites via Compression Test

**DOI:** 10.3390/ijms23116338

**Published:** 2022-06-06

**Authors:** Siti Humairah Kamarul Bahrain, Nor Nabilah Che Abd Rahim, Jamaluddin Mahmud, M. N. Mohammed, S. M. Sapuan, R. A. Ilyas, Samah Elsayed Alkhatib, M. R. M. Asyraf

**Affiliations:** 1 School of Mechanical Engineering, College of Engineering, Universiti Teknologi MARA, Shah Alam 40450, Malaysia; cnornabilah@gmail.com (N.N.C.A.R.); jm@uitm.edu.my (J.M.); 2 Mechanical Engineering Department, College of Engineering, Gulf University, Sanad 26489, Bahrain; dr.mohammed.alshekhly@gulfuniversity.edu.bh; 3 Laboratory of Biocomposite Technology, Institute of Tropical Forestry and Forest Products (INTROP), Universiti Putra Malaysia, Serdang 43400, Malaysia; sapuan@upm.edu.my; 4 Advanced Engineering Materials and Composites Research Centre (AEMC), Department of Mechanical and Manufacturing Engineering, Faculty of Engineering, Universiti Putra Malaysia, Serdang 43400, Malaysia; 5 School of Chemical and Energy Engineering, Faculty of Engineering, Universiti Teknologi Malaysia, Johor Bahru 81310, Malaysia; 6 Centre for Advanced Composite Materials (CACM), Universiti Teknologi Malaysia, Johor Bahru 81310, Malaysia; 7 Department of Mechanical Engineering, Faculty of Engineering & Technology, Future University in Egypt, New Cairo 11845, Egypt; samah.elmetwally@fue.edu.eg; 8 School of Mechanical Engineering, Faculty of Engineering, Universiti Teknologi Malaysia, Johor Bahru 81310, Malaysia; asyrafriz96@gmail.com

**Keywords:** silicone biocomposite, compression test, hyperelastic model, bamboo fibre, cellulosic fibres

## Abstract

Materials that exhibit highly nonlinear behaviour are intricate to study. This is due to their physical properties, as they possess a very large deformation. Silicone rubber is among the materials that can be classified as possessing such characteristics, despite their being soft and frequently applied in medical applications. Due to their low mechanical properties, however, it is believed that a filler addition could enhance them. This study, therefore, aims to investigate the effect of the addition of bamboo cellulosic filler to silicone rubber in terms of its compressive properties in order to quantify its material constants using the hyperelastic theory, specifically the Neo-Hookean and Mooney–Rivlin models. The specimens’ compressive properties were also compared between specimens immersed in seawater and those not immersed in seawater. The findings showed that the compressive properties, stiffness, and compressive strength of the bamboo cellulosic fibre reinforced the silicone rubber biocomposites, improved with higher bamboo filler addition. Specimens immersed in seawater showed that they can withstand a compressive load of up to 83.16 kPa in comparison to specimens not immersed in seawater (up to 79.8 kPa). Using the hyperelastic constitutive models, the Mooney–Rivlin model displayed the most accurate performance curve fit with the experimental compression data with an R^2^ of up to 0.9999. The material constant values also revealed that the specimens immersed in seawater improved in stiffness property, as the C_1_ material constant values are higher than for the specimens not immersed in seawater. From these findings, this study has shown that bamboo cellulosic filler added into silicone rubber enhances the material’s compressive properties and that the rubber further improves with immersion in seawater. Thus, these findings contribute significantly towards knowledge of bamboo cellulosic fibre–reinforced silicone rubber biocomposite materials.

## 1. Introduction

Moving towards a sustainable future, huge efforts have been made in raising awareness of environmental issues. Each day our world is getting hotter as global climate change progresses. This is obvious from the many recent forest fires, such as in the Amazon and Indonesia, where fire was triggered by the high temperature in the air. Moreover, this condition also caused the extinction of wildlife that live in the forests. This also happened to archaic arctic animals such as polar bears due to the ice melts caused by the global warming phenomenon. With these worrisome issues, green and sustainable energy has been widely explored by researchers to help preserve the earth [1,2,3,4,5].

It is known that one of the contributing factors to global climate change is the massive emission of carbon dioxide gases. One of the sources is the burning of synthetic fibres such as carbon fibre, glass fibre, and aramid, which are widely used in the making of composite materials [6,7,8,9,10,11,12]. These composite materials are also growing in scale, especially in automotive, aerospace, construction, and marine sectors, owing to their light weight, high strength, good corrosion resistance, and other qualities [13,14,15,16,17,18,19,20]. However, since they also cause side effects to the environment, researchers aimed to find an alternative to replace these synthetic fibres [21,22,23,24]. There have been many efforts, and studies are being conducted to discover the potential of natural fibres as a reinforcement in composite materials [25,26,27]. Such fibres are being reinforced into many types of hard matrices such as epoxy [28], polylactide, and polypropylene [28]. Examples of natural fibres are flax, kenaf, *Arenga pinnata*, sisal, wood, oil palm, and bamboo [29,30,31,32,33]. However, few studies have concentrated on a soft-type matrix, e.g., silicone rubber. In this study, bamboo fibre is chosen as a reinforcing material using silicone rubber as a matrix. Bamboo fibre is one of the natural fibres currently gaining attention by researchers since it is a fast growing plant [34,35,36]. In Malaysia, bamboo is widely employed in furniture manufacture, architectural design, the making of rafts, handicrafts [37], and food preparations (lemang) [38]. This is owing to bamboo’s versatility, durability, and safety for use in daily applications [39]. It is also proven to be good in medical applications, where in a previous study, Shanmugasundaram and Gowda [40] found that bamboo cellulosic fibres use to produce baby diapers showed a 100% and 98.75% reduction in antibacterial activities against the *E-coli* and *S.aureus bacteria*, respectively. Moreover, Singla et al. [35] attempted to use leaves of the bamboo species, *Dendrocalamus hamiltonii* and *Bambusa bambos*, for cellulose nanocystals (CNCs) isolation in wound dressing applications. It was found that CNCs are suitable to be developed for wound dressings as they improved in vivo skin tissue repair and regeneration.

Silicone rubber, on the other hand, is widely employed in medical sectors, especially for catheters, contact lenses, heart pacemakers, and blood oxygenator membrane, due to its good oxygen and carbon dioxide permeability [41,42]. Due to the soft property of silicone rubber, hyperelastic constitutive models have been adopted to characterize its mechanical properties [43,44]. However, few studies have adopted hyperelastic constitutive models in the field of natural fibres–reinforced silicone rubber biocomposites, and very few studies have quantified their hyperelastic material constants. Bahrain and Mahmud [45] reported on the tensile properties of a natural fibre called Arenga pinnata fibre–reinforced silicone rubber biocomposite using the Neo-Hookean model to acquire its material constant, C_1_. Their study found that the Neo-Hookean model could mimic the linear pattern of the 16 wt% specimens as compared to 8 wt% and 0 wt% specimens. With the same type of biocomposite material, the authors also performed another study by investigating its compressive properties for sealing application [46]. It was discovered that the *Arenga pinnata*–silicone biocomposite exhibited good sealing capability when soaked in seawater, as the specimens could withstand up to 70 kPa compressive load. The compressive behavior of the specimens was then analysed using the Neo-Hookean and Mooney–Rivlin models, and the latter has accurately described the non-linear properties of the soft biocomposite material. A study by Noor et al. [47] analysed the synthesisation of kenaf powder into silicone rubber and showed that the increasing values of Neo-Hookean and Mooney–Rivlin hyperelastic constants contribute to the stiffness properties of the composites. Azmi et al. [48] also investigates the tensile properties of kenaf silicone rubber biocomposite using the Neo-Hookean, Mooney–Rivlin, and Ogden models. Similarly, they reported that the increment of fibre content resulted in an increase in the hyperelastic material constants value. They also revealed that the Ogden model mimics the tensile behavior of the kenaf–silicone biocomposite better than the Neo-Hookean and Mooney–Rivlin models. A biocomposite material using agar as the filler for silicone rubber was also explored [49]. The authors concluded that the hyperelastic behavior of the agar-reinforced silicone rubber biocomposite material could deform almost similarly to the properties of human skin in terms of its tensile properties. This study could contribute significantly to research on skin substitutes, especially in wound healing.

Having assessing a few previous studies, we intend in the present study to introduce new bamboo fibre–reinforced silicone rubber biocomposite material for insole applications owing to the good antibacterial activities of both bamboo fibre and silicone rubber. Thus, preliminary studies have to be conducted since there is a lack of research investigating the mechanical properties of bamboo fibre–reinforced silicone rubber biocomposite and its potential as an insole. Insoles are placed at the bottom of our feet, whereby our body weight and pressure are subjected directly to the surface as we stand and walk. Moreover, it is undeniable that insoles are always exposed to wet conditions such as sweat and rain. It is essential for this new biocomposite material to be examined under extreme conditions, e.g., seawater [50]. Therefore, this study aims to assess for the first time the compressive properties of bamboo fibre–reinforced silicone rubber biocomposites compared to specimens variously immersed and not immersed in seawater. Due to its soft property and high deformation behaviour, it is assumed that the bamboo fibre–reinforced silicone rubber biocomposites material is hyperelastic, isotropic, and incompressible. Its material constant parameters are quantified using hyperelastic constitutive models, namely the Neo-Hookean and Mooney–Rivlin.

## 2. Results

### 2.1. Compressive Properties

**Figure 1:**
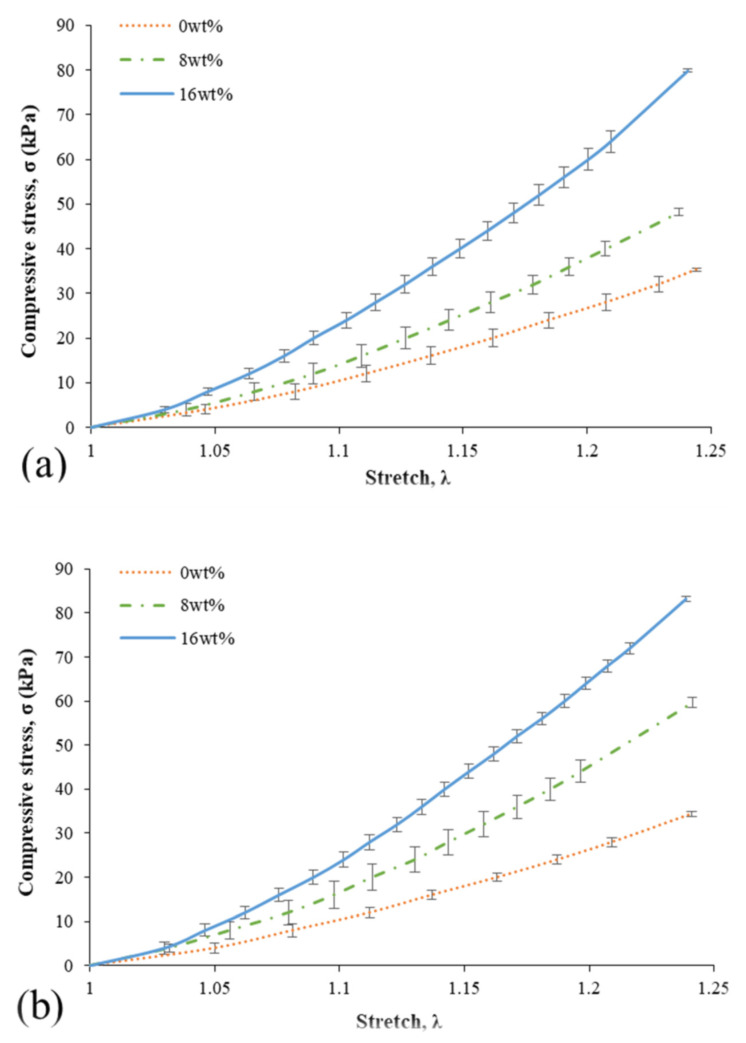
The average compressive strength of composites (**a**) without being immersed and (**b**) immersed in seawater.

Figure 1a,b displays the compressive behaviour of bamboo fibre–reinforced silicone rubber biocomposites for 0, 8 and 16 wt% specimens where comparison was made between the composites immersed and not immersed in seawater. The *x* and *y*-axes of Figure 1 represent the stretch, λ; and compressive stress, σ, respectively. From the figure, both results display the deformation behaviour of bamboo fibre–reinforced silicone rubber biocomposites and exhibited a nonlinear elastic curve where compressive load increases nonlinearly with the increase of stretch. Moreover, both the specimens without immersion (Figure 1a) and those under immersion (Figure 1b) in seawater revealed that the further addition of bamboo fillers increases the stiffness of the composites. 

**Figure 2:**
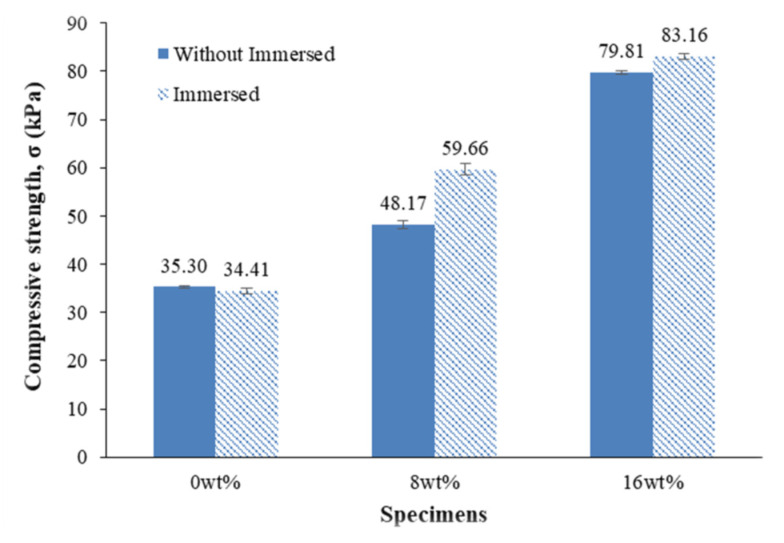
Comparison of the average ultimate compressive strength of bamboo fibre–reinforced silicone rubber biocomposites among specimens with and without immersion in seawater.

Figure 2 illustrates the average ultimate compressive strength of bamboo fibre–reinforced silicone rubber biocomposites compared to specimens with and without immersion in seawater. It was observed that the effect of immersion of the specimens into the seawater improves their compressive strengths. 

### 2.2. Hyperelastic Material Constants

**Figure 3:**
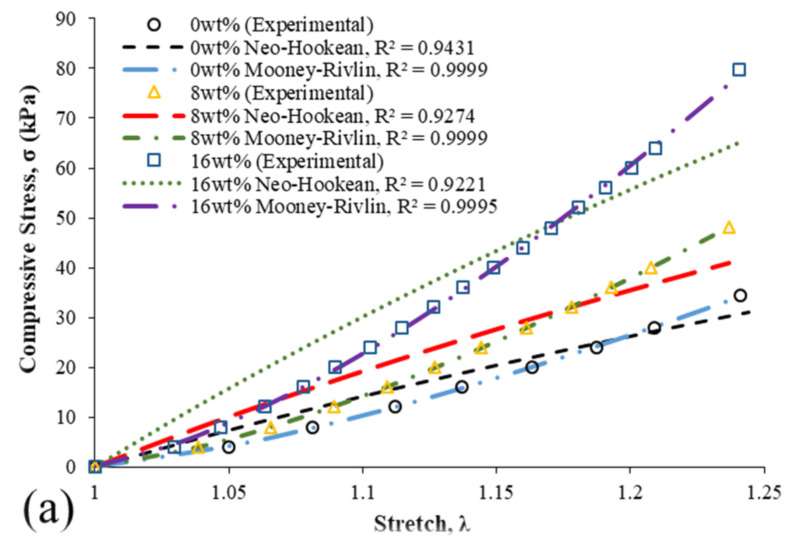

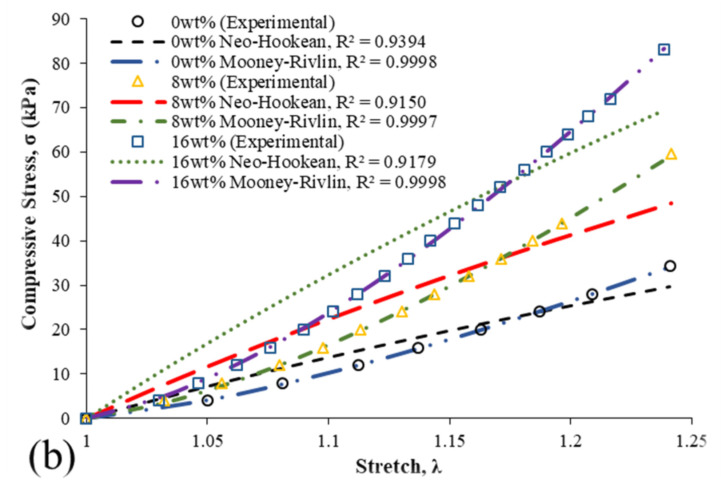
Hyperelastic curve fittings of specimens (**a**) without being immersed and (**b**) immersed in seawater.

The hyperelastic analyses of bamboo fibre–reinforced silicone rubber biocomposites of 0, 8, and 16 wt% specimens are presented in Figure 3. From these figures, the Neo-Hookean and Mooney–Rivlin models were adopted for both specimens without immersion (Figure 3a) and with immersion (Figure 3b) in seawater. It was observed that the Mooney–Rivlin model shows the best performance in accurately mimicking all experimental compressive curves of the bamboo fibre–reinforced silicone rubber biocomposites with an R^2^ of up to 0.9999 in comparison to the Neo-Hookean model. The Neo-Hookean model seems to exhibit a similar trend for all specimens, as it behaves with a concave downward pattern. 

Table 1 and Table 2 show the hyperelastic material constant values obtained for 0, 8, and 16 wt% specimens without being immersed and immersed in seawater, respectively. From these tables, it can be seen that the Neo-Hookean parameter C_1_ increases with the increase in filler contents. However, these C_1_ values for 0, 8, and 16 wt% are inaccurate to describe the deformation behaviour of the bamboo fibre–reinforced silicone rubber biocomposites due to the weak curve fitting ability as depicted in Figure 3. On the other hand, the Mooney–Rivlin material constants, C_1_ and C_2_, show increasing and decreasing trends with the increment of filler contents. 

## 3. Discussion

From the compression tests, the 0 wt% specimens showed the lowest steepness of the graph and hence the lowest stiffness value. This property was further improved for 8 and 16 wt% specimens as the latter displayed the highest stiffness property. The 16 wt% specimens also possessed the highest compressive load as compared to the 8 wt% specimens, followed by the 0 wt% specimens. This indicates that the addition of fillers into the silicone rubber improved the compressive properties of the composites in withstanding the compressive load and in resisting deformation due to the applied load [51]. This could also be attributed to an excellent filler–polymer interaction [52], as the fibres are well distributed. With this, the stress distribution and deformation of the specimens will be uniformly transferred, and thus ultimate compressive performance of the specimens can be achieved [53].

The increasing compressive strength may be attributed to the good interfacial adhesion between the fibre and the matrix as it promotes good bonding. This is in agreement with the previous findings conducted by Amatosa et al. [54] where their seawater-treated laminated bamboo composites were superior in compressive and bending strengths compared to the untreated ones. As the specimens were exposed to the seawater, the outer layer of the fibres was removed owing to the salinity of the seawater, thus increasing their durability to withstand more loads [55]. In comparison to specimens without the exposure of seawater, the presence of the outer layer of the bamboo fibres causes weak adhesion between the fibre and the matrix. This resulted in the decrease in their durability and ability to withstand the compressive load received from the silicone rubber. Moreover, the mobility of the polymer chain was restricted due to the good fibre–matrix bonding. This increases the strength and hardness of the composite [56]. This has shown that the addition of fillers into silicone rubber could improve significantly with the stiffness of the specimens as the polymeric chain of the silicone rubber has been altered by the presence of the fillers.

With only one material constant (C_1_) parameter, the hyperelastic findings show that the Neo-Hookean model is weak to perform and mimic a highly nonlinear deformation behaviour with an R^2^ of up to 0.9431. This is due to the simplicity of the Neo-Hookean model, and it can make the best approximation at a relatively low strain condition [57]. Several other studies reported similar findings where Kim et al. [58] revealed that the Neo-Hookean model could only describe the tensile behaviour of chloroprene rubber below the 100% strain range. The Neo-Hookean model could only be adopted for low-to-moderate strain deformation behaviour materials, as it has weak capability to predict the material’s behaviour at a larger deformation [59,60]. These outcomes suffice to clarify that the Neo-Hookean model is inaccurate to describe a highly nonlinear elastic behaviour, as it only could predict accurately at a low deformation behaviour.

It could also be said that with the C_1_ parameter values for 0, 8, and 16, wt% are increased; this indicates that the stiffness property of the composites is increased. This stiffness property has also been proven via the compression test, as further addition of bamboo fibres into the silicone rubber increases the stiffness of the specimens. As for the C_2_ material constants, the decrement trend could describe that the linearity of the specimen is decreasing, and thus these C_1_ and C_2_ values could make the best fit curve to mimic the materials’ deformation behaviour. In comparison, it can be observed that the material constant values for the specimens immersed in seawater are higher than for the specimens not immersed in seawater. This finding via hyperelastic studies further supports that the stiffness property of the specimens is improved as compared to the specimens not immersed in seawater. There are several other reported findings on the values obtained by adopting the Mooney–Rivlin model in the study. An example is provided by L. Meunier et al. [43] in their study on unfilled silicone rubber undergoing several tests including a compression test. The obtained material constants values, C_1_ and C_2_, were 0.14 MPa and 0.023 MPa, respectively. P. Huang et al. [61] investigated rubber seal strip under a compression test and found that the Mooney–Rivlin material constants value obtained was 1.015 MPa and 0.145 MPa for C_1_ and C_2_, respectively. In a study by Huri and Mankovits [62], the fitted Mooney–Rivlin material constants C_1_ and C_2_ for styrene–butadiene rubber (SBR) were 1.28801 MPa and 1.1371 MPa, respectively. From these reported values, it can be compared that the bamboo fibre–reinforced silicone rubber biocomposite material is comparable to other rubber-like materials. It can also be seen the current studies display slightly lower values than [61,62], which indicates that this new biocomposite material is soft and suitable for insole applications.

## 4. Materials and Methods

### 4.1. Materials

The type of bamboo chosen for this study is known as *Gigantochloa albociliata* and was purchased from local people in Rawang, Selangor, Malaysia. For the matrix, we used the Silicone Ecoflex 00-30 platinum cure supplied by Castmech Technologies Sdn Bhd, Ipoh, Perak, Malaysia. The product is composed of two parts, Part A and Part B, and both were mixed thoroughly by the ratio of 1:1.

### 4.2. Specimen Preparation

The bamboo stalks were cut into smaller pieces and dried using the oven for 24 h at 70 °C to remove the moisture. The stalks were then cooled at room temperature. Once fully cooled, the cut bamboo stalks were crushed using a jaw crusher and followed by the planetary mono mill for further refinement into particulate bamboo fillers. Finally, the bamboo fillers were sieved using a sieve frame mesh of 250 μm size.

With 0 (pure silicone rubber), 8, and 16 wt% filler contents, the weighted filler was added into a well-hand-stirred silicone rubber solution. The stirring process was continued, and it was ensured that the sides of the container were fully scraped down to obtain a homogenous specimen. Finally, the mixture was poured into the mould and let to cure at room temperature for at least four hours. These procedures were repeated for the next batch of specimens, which were immersed in seawater for seven days.

### 4.3. Compression Tests

**Figure 4:**
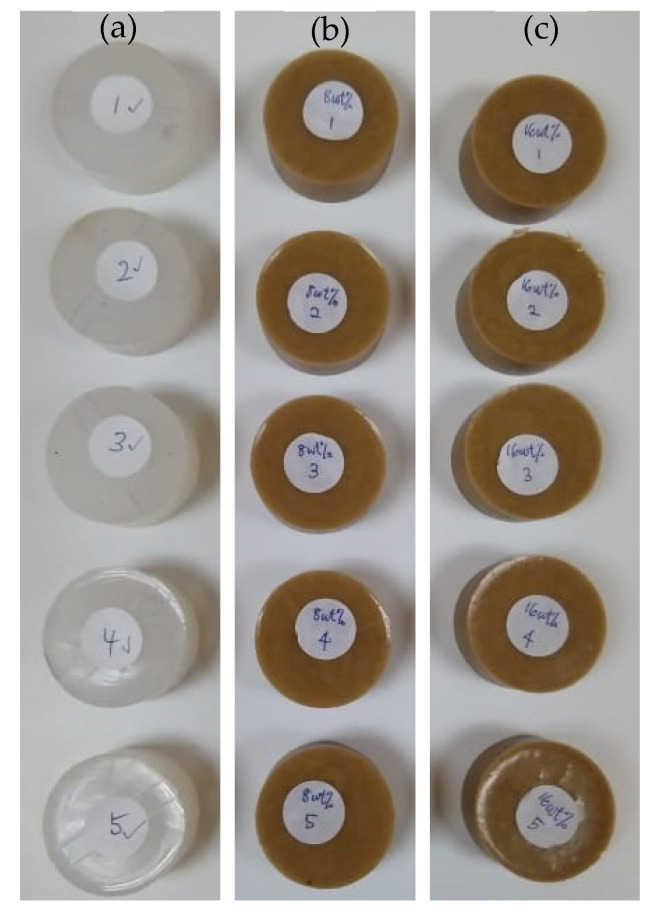
(**a**) 0 wt%, (**b**) 8 wt%, and (**c**) 16 wt% bamboo fibre–reinforced silicone rubber biocomposite specimens for compression test.

The compressive properties of bamboo fibre–reinforced silicone rubber biocomposites were investigated under a compression testing machine using Instron Universal Testing Machine at the Strength of Materials Laboratory, School of Mechanical Engineering, College of Engineering, Universiti Teknologi MARA, Shah Alam, Selangor, Malaysia. The ASTM D575 standard was used for this test [63]. The specimens were prepared with a dimension of 12.9 mm in thickness and a diameter of 29 mm (Figure 4). The specimens with and without being immersed in seawater were then compared. From these tests, compressive stresses, σ—strains, and ε curves were obtained. To ensure consistency and repeatability, the average compressive properties from five specimens of each of the filler contents were recorded.

### 4.4. Quantifying Hyperelastic Material Constants

The selected hyperelastic constitutive models in this study are the Neo-Hookean and Mooney–Rivlin models. Their strain energy density functions were expressed as in Equations (1) and (2), respectively:

W = C_1_ (I_1_ − 3) (1)

W = C_1_ (I_1_ − 3) + C_2_ (I_2_ − 3) (2)

where the I_1_ and I_2_ are the strain invariants.

Considering bamboo fibre–reinforced silicone rubber biocomposite specimens to be incompressible, hyperelastic, and isotropic, the Neo-Hookean and Mooney–Rivlin models are expressed in terms of engineering stress, σ, and stretch, λ as shown in Equations (3) and (4).

(3) $$\sigma_{E}\;=2C_{1}\;\left(\lambda -\frac{1}{\lambda^{2}}\right)$$

(4) $$\sigma_{E}\;=2C_{1}\;\left(\lambda -\frac{1}{\lambda^{2}}\right)+2C_{2}\;\left(1-\frac{1}{\lambda^{3}}\right){\;}_{\;}$$

The compressive stress, σ—strain, and ε data obtained earlier were converted into compressive stress, σ—stretch, and λ relation, where the stretch, λ values were computed using Equation (5).

*λ* = 1 + ε (5)

In order to perform the curve fittings and acquire the material constants (C_1_ for the Neo-Hookean and C_1_ and C_2_ for the Mooney–Rivlin models) values, Excel Solver add-in was used. A simple regression method was performed using the square of the error formula as shown in Equation (6).

E = (Predicted value − Experimental value)^2^ (6)

Finally, the quality of fit between the experimental data and the hyperelastic models for each specimen was calculated using the coefficient of determination R^2^, where R^2^ = 1 would be a perfect fit.

## 5. Conclusions

This study investigated the compressive properties of bamboo fibre–reinforced silicone rubber biocomposites with a filler content of 0, 8, and 16 wt%. A comparison between specimens not immersed and immersed in seawater was successfully conducted. The results revealed that further addition of bamboo filler contents enhanced the compressive properties of the biocomposite materials. The specimens immersed in seawater also showed superior compressive strength compared to the specimens not immersed in seawater. By adopting the Neo-Hookean and Mooney–Rivlin models in quantifying the material constant values, the Mooney–Rivlin model showed the best performance to curve fit for the experimental data with an R^2^ of up to 0.9999 in comparison to the Neo-Hookean model with an R^2^ of up to 0.9431. The Mooney–Rivlin material constant C_1_, which indicates its stiffness property, also revealed that specimens immersed in seawater have higher values as compared to specimens not immersed in seawater. Hence, this study has successfully determined the compressive properties of bamboo fibre–reinforced silicone rubber biocomposites and demonstrated them using hyperelastic constitutive models. These new findings could contribute to more research on bamboo fibre–reinforced silicone rubber biocomposite as a potential application for insoles, in addition to promoting more environmentally friendly materials.

## Figures and Tables

**Table 1:** Neo-Hookean and Mooney–Rivlin Material Constants (kPa) for specimens without being immersed in seawater.

Specimen (wt%)	Material Constants (kPa)		
	Neo-Hookean	Mooney-Rivlin	
	C_1_	C_1_	C_2_
0	25.965	107.499	−97.281
8	35.004	163.987	−151.779
16	55.074	261.279	−241.705

**Table 2:** Neo-Hookean and Mooney–Rivlin Material Constants (kPa) for specimens immersed in seawater.

Specimen (wt%)	Material Constants (kPa)		
	Neo-Hookean	Mooney-Rivlin	
	C_1_	C_1_	C_2_
0	25.143	107.434	−97.455
8	40.865	202.583	−189.399
16	59.054	288.867	−269.905

## Data Availability

Not applicable.
